# South African flag sign to a giant coronary artery aneurysm

**DOI:** 10.1093/ehjcr/ytae028

**Published:** 2024-02-01

**Authors:** Rakavi Rathinasamy, Nirmal Ghati, Neeraj Parakh, Sanjeev Kumar, Akshay Kumar Bisoi, Sudheer Arava, Rajiv Narang, Balram Bhargava

**Affiliations:** Department of Cardiology, All India Institute of Medical Sciences, New Delhi 110029, India; Department of Cardiology, All India Institute of Medical Sciences, New Delhi 110029, India; Department of Cardiology, All India Institute of Medical Sciences, New Delhi 110029, India; Department of Cardiac Radiology, All India Institute of Medical Sciences, New Delhi, India; Department of Cardiothoracic Vascular Surgery, All India Institute of Medical Sciences, New Delhi, India; Department of Pathology, All India Institute of Medical Sciences, New Delhi, India; Department of Cardiology, All India Institute of Medical Sciences, New Delhi 110029, India; Department of Cardiology, All India Institute of Medical Sciences, New Delhi 110029, India

**Keywords:** IgG4-related disease, Coronary artery aneurysm, STEMI, South African flag sign

## Abstract

**Background:**

Coronary arteritis leading to aneurysm is one of the unusual presentations of IgG4-related disease. Acute myocardial infarction as a complication of IgG4-related giant coronary artery aneurysm is even rarer.

**Case summary:**

We describe the case of a 56-year-old gentleman who presented to our institute with Canadian Cardiovascular Society (CCS) class III angina. His symptoms were persistent even with high-dose antianginal medications. He had an acute coronary syndrome two weeks back for which he was treated conservatively in a peripheral health centre. His 12-lead electrocardiogram at the time of the event was suggestive of high lateral ST-segment elevation myocardial infarction (South African flag sign). His transthoracic echocardiography showed mild left ventricular dysfunction and a large echogenic mass lateral to the left ventricle. Coronary angiography followed by cardiac computed tomography revealed a giant pseudoaneurysm of the proximal and mid-left anterior descending coronary artery. FDG-PET scan showed significant metabolic activity in the aneurysm wall and mediastinal lymph nodes suggesting active inflammation. IgG4-related coronary arteritis was suspected, and the patient underwent aneurysmectomy and coronary artery bypass (CABG) surgery. The histopathology of the resected segment showed diffuse IgG4-secreting plasma cells confirming the diagnosis.

**Discussion:**

Atherosclerosis is the most common cause of coronary aneurysms in adults. However, cardiologists should be aware of atypical causes like IgG4-related disease that can even present with acute coronary syndrome. Although multimodality imaging is beneficial during early evaluation, histopathological analysis is the cornerstone for the diagnosis of IgG4-related disease. The management involves both immunosuppressive medication and endovascular or surgical repair.

**Learning points**

- To know about the significance of the ‘South African flag sign’ in a 12-lead ECG.
- To learn about the differential diagnosis of the coronary aneurysms.
- To learn pathophysiology and clinical presentations of IgG4-related coronary arteritis and aneurysm.

## Primary specialities involved other than cardiology

Radiology, Nuclear medicine, Pathology, Cardiothoracic surgery.

## Introduction

Coronary artery aneurysm (CAA) is defined as focal dilatation of the coronary artery of at least 1.5 times the normal adjacent segment. A similar lesion with >8 mm luminal diameter or >4 times dilatation than the healthy segment is labelled as giant CAA. The reported prevalence of CAA is 1.4% and giant CAA is even rarer (0.02%). Right coronary artery (40.4%) is the most involved artery followed by left anterior descending (32.3%), left circumflex (23.4%), and left main (3.5%) arteries.^1^ Atherosclerosis and Kawasaki disease are the predominant aetiology in adults and paediatric populations, respectively. Other notable causes are systemic vasculitis, congenital, iatrogenic, cocaine use, and infective. The exact pathophysiology is incompletely understood. However, the hypothesis is that an elevated concentration of proinflammatory cytokines and matrix metalloproteinases (MMPs) in the diseased vessel wall destroys the arterial media. Gradually, the arterial wall is thinned out resulting in the formation of aneurysms.^2^ We present a case of giant CAA due to IgG4-related coronary arteritis who suffered an acute coronary event (ACS) with ‘South African flag sign’ in the corresponding 12-lead electrocardiogram (ECG).

## Summary figure

**Figure:**
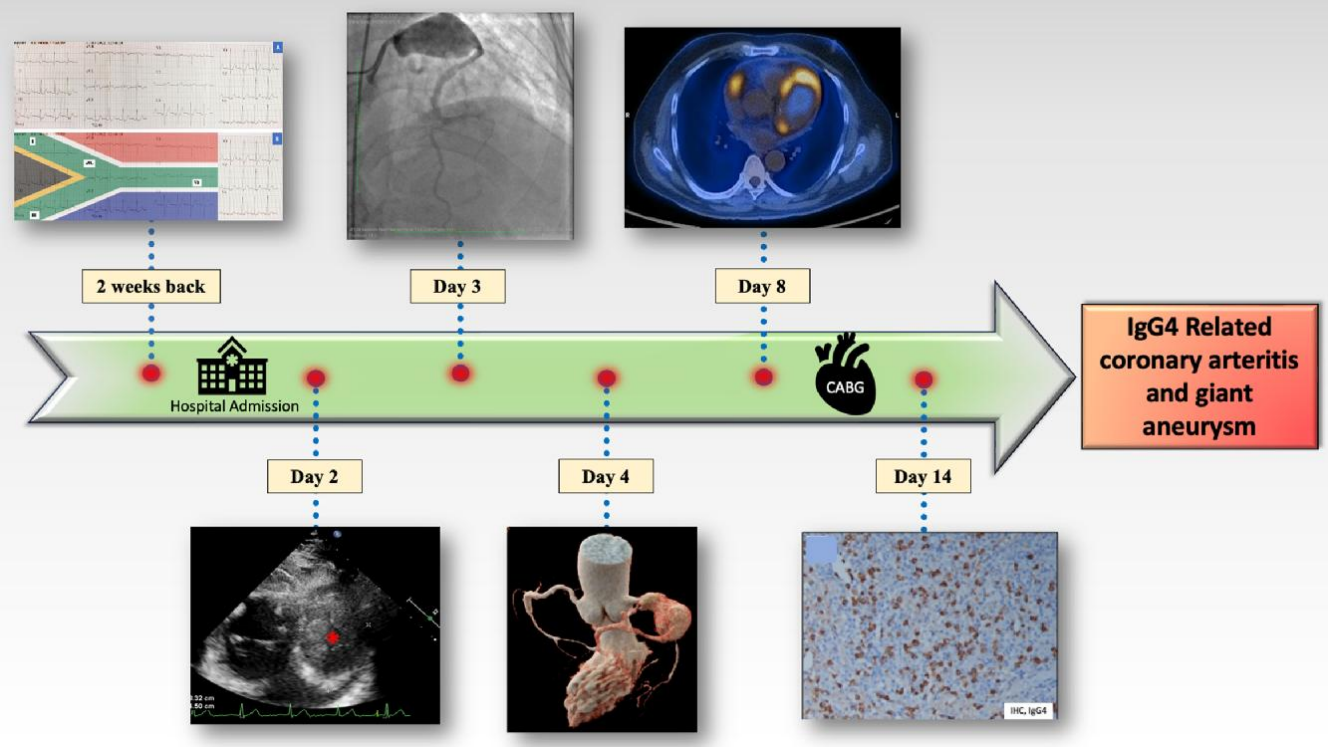


## Case presentation

**Figure 1:**
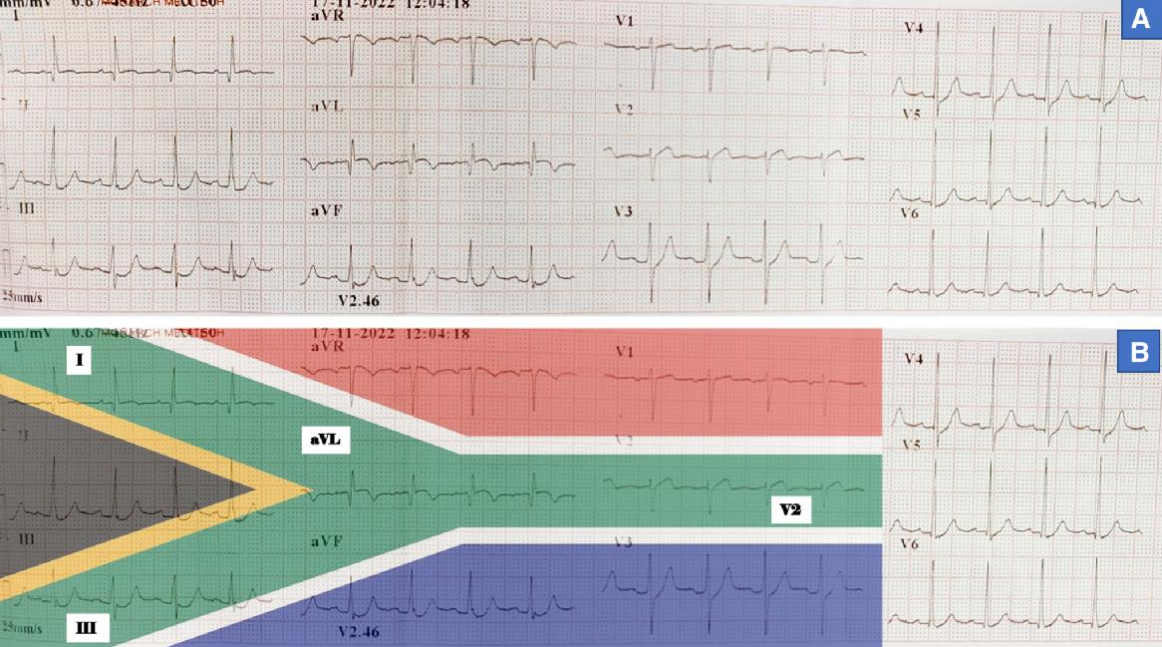
12-Lead electrocardiogram (ECG) at the time of acute coronary syndrome (ACS). (*A*) ECG showed ≥1 mm ST-segment elevation in leads I, aVL, V2 and ≥1 mm ST depression in lead III; (*B*) ‘South African flag sign’ suggestive of high lateral wall myocardial infarction.

A 56-year-old gentleman presented to our institute with Canadian Cardiovascular Society (CCS) class III angina for two weeks. His symptoms were persistent even with aspirin, clopidogrel, high-dose statin, and high-dose antianginal medications. He had a history of an ACS two weeks back for which he received conservative therapy in a peripheral health centre. His ECG at the time of ACS was suggestive of ‘South African flag sign’ (≥1 mm ST-segment elevation in leads I, aVL, V2, and ≥1 mm ST depression in lead III) (*Figure 1*) indicating high lateral ST-segment elevation myocardial infarction (STEMI). At that time, he was not revascularized by thrombolysis or primary angioplasty and was later referred to us with persistent angina. At presentation, his blood pressure and heart rate were 128/76 mmHg and 96/min, respectively. General physical examination revealed pallor. His jugular venous pressure was normal and there was no abnormal heart sound or cardiac murmur. Rest of the systemic examination was also normal.

He had psoriatic skin lesions on the extensor aspect of both forearms for the past four years. He also had history of two transient ischaemic attacks (TIAs) 10 years back. He quit smoking after TIAs and had no major cardiovascular disease risk factors. There was no family history of coronary artery disease, sudden cardiac death, and autoimmune disease. The patient belongs to lower-middle socioeconomic class and did not have any major psychiatric illness. There was no history of any illicit drug abuse.

His baseline complete blood count showed severe anaemia with haemoglobin level of 8.5 g/dL. Peripheral smear showed normocytic normochromic red blood cells suggesting anaemia due to chronic inflammation. Rest of the blood counts, kidney function tests, and liver function tests were normal. His baseline total cholesterol, LDL, and HDL levels were 178 mg/dL, 97 mg/dL, and 42 mg/dL, respectively, and his HbA1C was in the non-diabetic range. His transthoracic echocardiography revealed a huge echogenic mass at the lateral wall of the left ventricle and moderate pericardial effusion (see Supplementary material online, *Video S1*). Left ventricular ejection fraction was mildly impaired with hypokinesia of the mid-anterolateral, apical-lateral segments. Coronary angiography showed a giant coronary aneurysm in the proximal and mid-left anterior descending (LAD) artery, part of which was filling faintly in late frames due to a large peripheral thrombus (see Supplementary material online, *Videos S2*–*S4*). The rest of the LAD showed mild disease. The left circumflex artery (LCX) was normal. The right coronary artery (RCA) showed mid and distal ectatic segments with no significant obstruction. However, there was a delayed globular filling of contrast near the proximal RCA that could not be characterized properly with coronary angiography.

**Figure 2:**
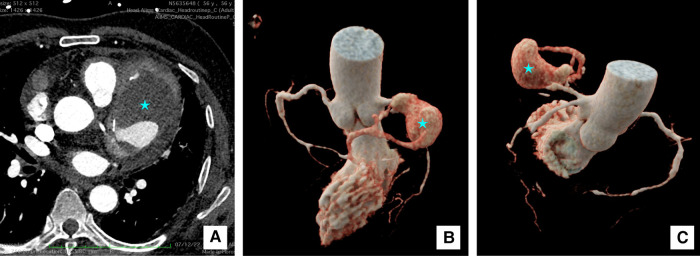
Cardiac computed tomography (CT) images. (*A*) 2D transverse section image at the level of aortic sinus showing giant saccular pseudoaneurysm of left anterior descending artery (*) measuring 8.1 × 5.2 × 4.7 cm with peripheral thrombus and calcific foci; (*B* and *C*) 3D reconstruction images of the pseudoaneurysm (*).

**Figure 3:**
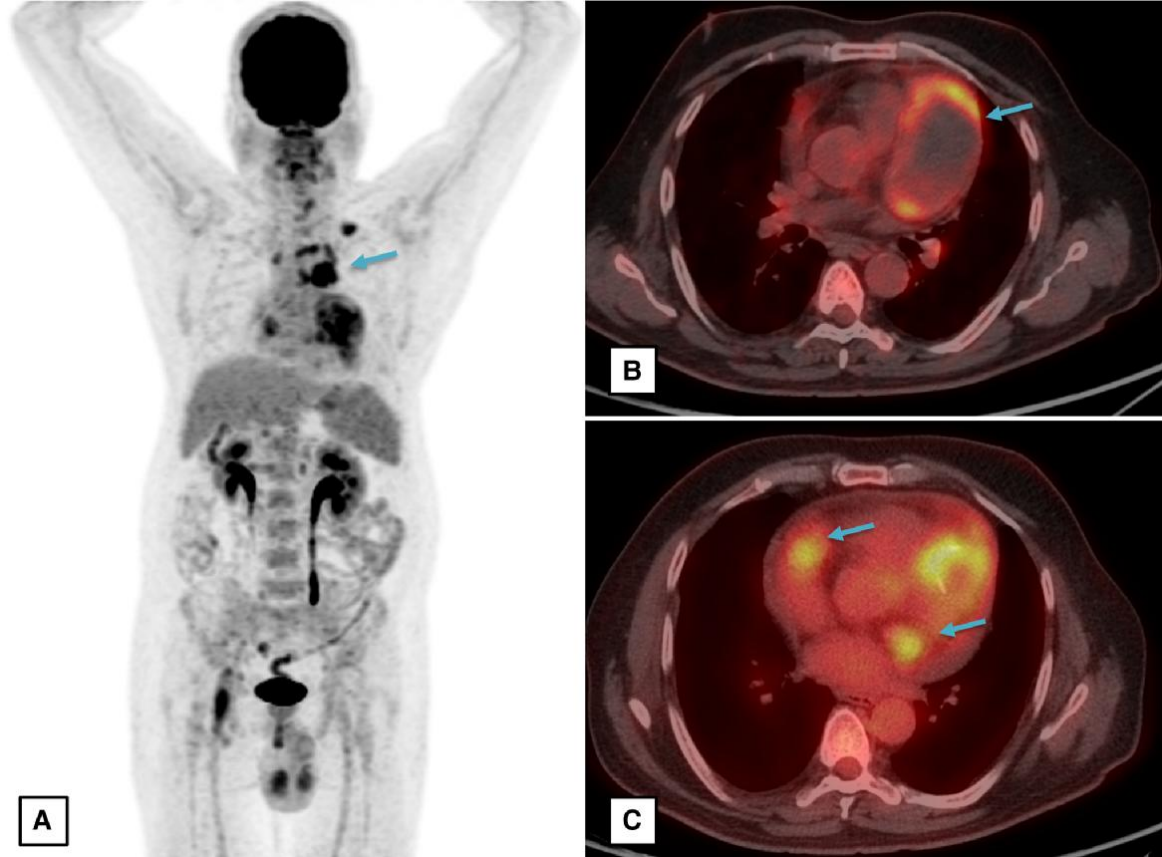
Fluorodeoxyglucose-positron emission tomography (FDG-PET) images. (*A*) Whole body coronal section, (*B* and *C*) transverse section at aortic sinus and cardiac level, respectively. Images showing significant FDG uptake (star *) in the perivascular soft tissue masses including the wall of left anterior descending (LAD) artery pseudoaneurysm.

A computed tomography (CT) coronary angiography was done for further clarification. It showed a giant pseudoaneurysm (measuring 8.1 × 5.2 × 4.7 cm) involving the proximal and mid-LAD artery (*Figure 2*). The aneurysm was partially filled with a large thrombus and causing a mass effect on the left pulmonary veins and main pulmonary artery. The arterial walls were thickened and enhanced. Moreover, there were multiple contrast-enhanced peri-coronary soft tissue lesions around the proximal and distal LCX, ostio-proximal obtuse marginal branch, and proximal RCA. Multiple large discrete homogenously enhancing mediastinal lymph nodes were seen in pre-vascular, left paratracheal, lower cervical, and superior mediastinum. His whole body ^18^F-fluorodeoxyglucose-positron emission tomography (FDG-PET) showed a large hypodense area and significant peripheral metabolic activity within the giant CAA suggestive of active vasculitis and central thrombosis. Multiple FDG avid upper paratracheal, paraaortic (largest ∼2.7 × 1.7 cm), and pre-vascular lymph nodes were also noted (*Figure 3*). There was no systemic involvement of other vessels or organs in the FDG-PET scan.

**Figure 4:**
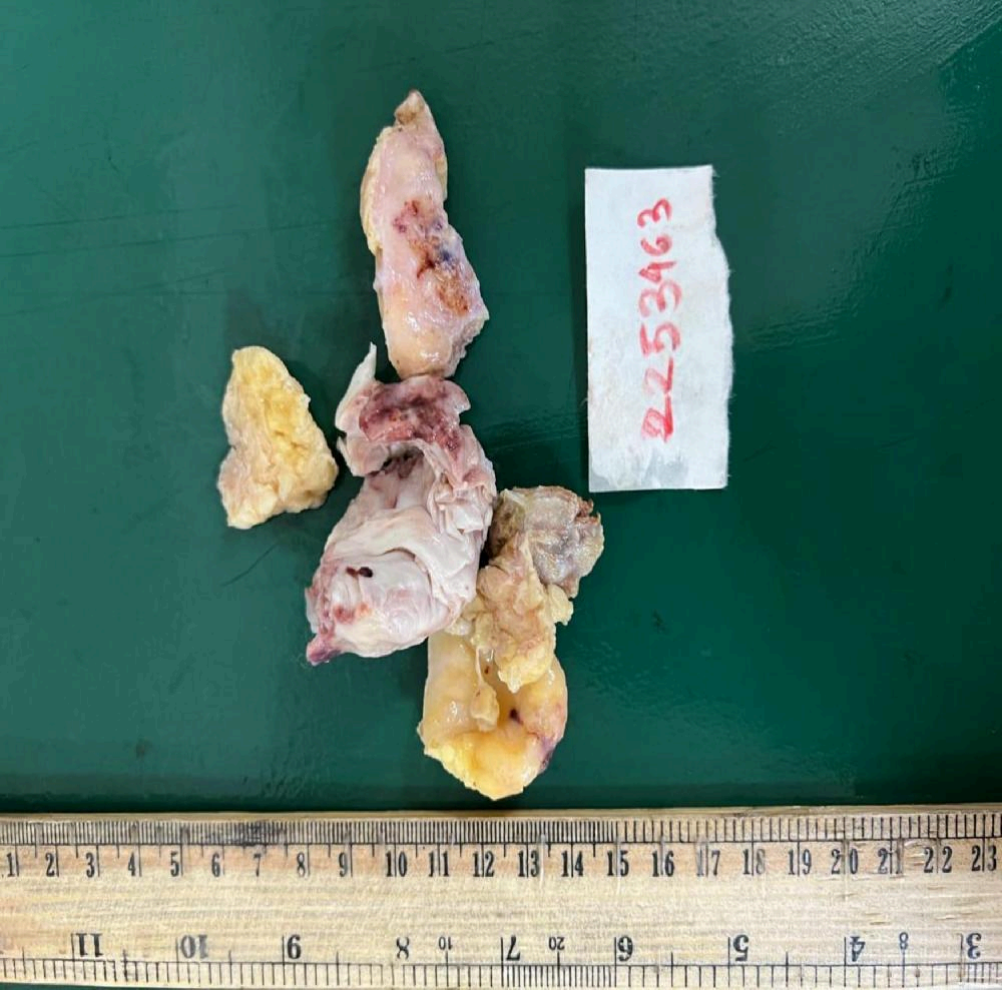
Picture of the resected specimens of the IgG4-related giant coronary artery.

**Figure 5:**
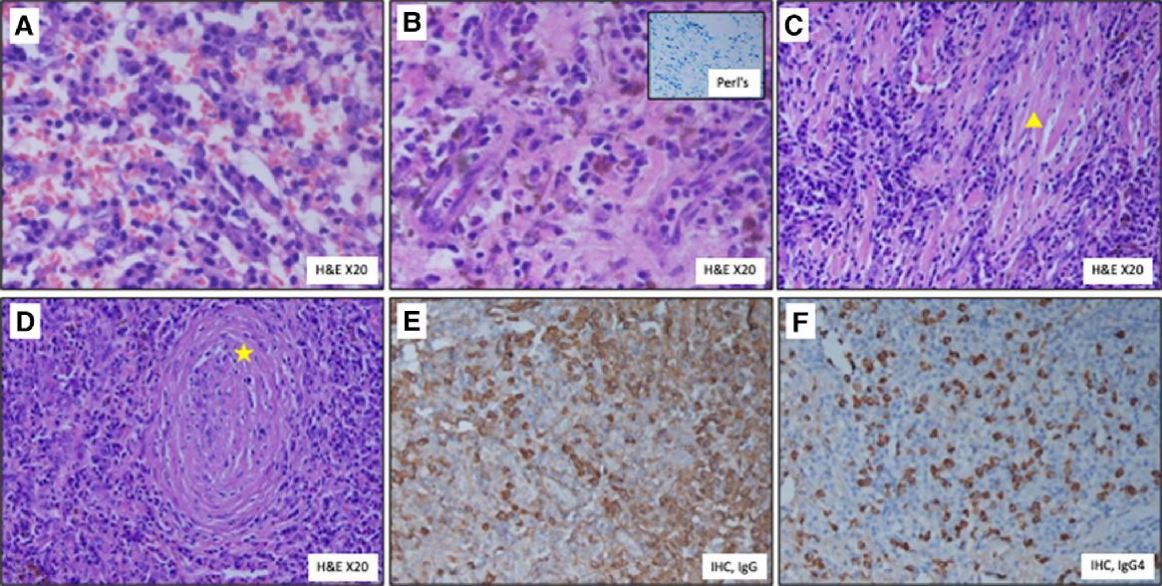
Histopathology of the resected aneurysm. (*A*) Diffuse infiltration of plasma cells; (*B*) haemosiderin pigment deposition [golden brown pigment]. Inset—Perl’s Prussian blue iron stain imparting blue colour to the deposited iron; (*C*) fibrosis (Δ); (*D*) obliterative phlebitis (star *); (*E*) IgG immunostain highlighting the IgG secreting plasma cells; (*F*) IgG 4 immunostain highlighting the IgG 4 secreting plasma cells. H&E, haematoxylin and eosin stain; IHC, immunohistochemical stain.

Giant coronary aneurysm, multiple hyper-enhancing soft tissue lesions whose appearance is characteristically called ‘pigs in the blanket’,^3^ multiple mediastinal and cervical lymph nodes, and significant FDG uptake in the lesions arose suspicion of IgG4-related disease. Chronic plaque psoriasis-like lesions are also one of the characteristic lesions seen in IgG4-related skin disorders.^4^ In addition, the serum IgG4 level was elevated (3.46 g/L; normal range: 0.05 to 1.25 g/L). Other diseases causing arteritis like antineutrophil cytoplasmic antibody (ANCA)-associated vasculitis, polyarteritis nodosa (PAN), giant cell arteritis, and Behcet’s disease were also kept as differential diagnoses. However, his antinuclear antibody (ANA) and ANCA tests were unremarkable. Also, his C-reactive protein level was 5.18 mg/L that is very unusual in patients with giant cell arteritis. His pathergy test was done to diagnose Behcet’s disease but it was negative. He also denied any history of illicit drug abuse such as cocaine that can cause CAAs. As there were severe ischaemic symptoms and a high risk of rupture of the giant pseudoaneurysm, the patient underwent LAD aneurysmectomy. Proximal LAD was closed with a PTFE graft and the right saphenous venous graft (RSVG) was anastomosed to the distal LAD. Similarly, RCA ectatic segment was excised and RSVG was anastomosed to the distal RCA. Internal mammary artery graft was not used to avoid IgG4-related arteritis in the graft vessels in the future. The resected segments were filled with eccentric thrombus (*Figure 4*). Histopathological examination of the resected aneurysmal wall revealed diffuse infiltration of plasma cells. Immunostaining revealed diffuse IgG4-secreting plasma cells (*Figure 5*). The IgG4/IgG ratio was 45–50% (normal value < 40%), which confirmed the diagnosis of IgG4-related coronary periarteritis and pseudoaneurysm.^5,6^

The post-operative course was uneventful. Aspirin, clopidogrel, high-dose statin, beta-blocker, and angiotensin-converting enzyme inhibitor (ACEI) were started during the post-surgical period. Because of biopsy-proven IgG4-related disease, oral steroid (prednisolone) was also started (0.5 mg/kg daily) two weeks after the surgery and it was continued for six weeks. Then it was tapered and stopped over two weeks duration. His ^18^F-FDG-PET and CT coronary angiography were repeated after the stoppage of steroid, and they did not reveal any recurrence of vasculitis or significant structural abnormality. The patient was asymptomatic at six months post-surgical follow-up.

## Discussion

IgG4-related disease is a fibroinflammatory condition characterized by the formation of mass-like lesions, intense lymphoplasmacytic infiltrate rich in IgG4-positive plasma cells, and storiform fibrosis causing systemic organ damage. The most involved organs are the biliary tree, salivary glands, periorbital tissues, kidneys, lungs, and lymph nodes.^7^ Large vessel vasculitis is the predominant IgG4-related cardiovascular disorder. It mostly affects elderly (52–81 years) males.^8^ IgG4-related medium vessel inflammation like coronary arteritis is rare and mostly reported as diffuse wall thickening (92%), stenotic (67%), aneurysmal (42%), and a combination of all three morphologies (22%).^8^

Myocardial infarction as a complication of IgG4-related coronary arteritis has been described in only a few case reports.^9,10^ Hence, a giant coronary aneurysm presenting with the ‘South African flag sign’ has made our case a unique one. The distinctive ECG sign was first described by Littmann^11^ to portray 12-lead ECG changes in high lateral STEMI. It is characterized by ST-segment elevation in leads I, aVL, V2, and ST depression in lead III. These non-contiguous ST changes in a 3 × 4 layout ECG strip align perfectly with the middle green (horizontal Y) zone of the South African flag (*Figure 1*). This atypical sign is often missed and might be the reason behind the inadequate treatment during ACS in our patient. Although our patient presented with ACS and persistent angina, his history of TIAs 10 years back was probably due to the same disease. This also reaffirms the variability in the presentation and the protracted course of the disease.

Since the symptoms are generally non-specific and subacute, IgG4-related periarteritis is mostly detected incidentally during imaging. Multiplanar contrast-enhanced CT is the investigation of choice to detect the exact extent of aneurysm; typical pseudotumor surrounding the vessels or other organ systems with contrast enhancement in the delayed phase. FDG-PET is helpful in assessing the inflammation that can be used to differentiate the disease from atherosclerosis-related coronary aneurysms.^12^ Serum IgG4 level is usually elevated in ∼70% of cases. Histopathological analysis of biopsy specimens is the gold standard for diagnosis. Elevated IgG4 and IgG-bearing plasma cells in tissues are prerequisite but non-specific findings. The ratio of IgG4-bearing plasma cells to IgG-bearing plasma cells (IgG4/IgG ratio) > 40% in immunohistochemical analysis confirms the diagnosis.^5–7^ Other differentials can be classified into three categories—atherosclerotic, inflammatory, and non-inflammatory.^13^ Atherosclerotic CAAs are more prone in males, affect older populations, and are associated with multiple risk factors like hypertension, diabetes, dyslipidaemia, and cigarette smoking. CT or invasive angiography usually shows multiple aneurysms involving more than one coronary artery in addition to areas of stenosis and evidence of atherosclerosis elsewhere. Diseases causing inflammatory CAA in adults are Behcet’s disease, PAN, granulomatosis with polyangiitis, microscopic polyangiitis, Takayasu arteritis, and giant cell arteritis. Behcet’s disease is a relapsing systemic vasculitis that involves veins and arteries of all sizes and causes aneurysm, stenosis, and mixed lesions. The classical symptoms are recurrent genital ulcers, aphthous oral ulcers, erythema nodosum, and ocular lesions like uveitis, and iridocyclitis. Budd–Chiari syndrome, multifocal parenchymal brainstem and cerebral involvement, and cerebral venous thrombosis are also reported. The disease usually manifests in men in the 3rd and 4th decades of life. Cardiac involvement is variable and includes pericarditis, myocarditis, endocarditis, intracardiac thrombus, and coronary vasculitis. CT coronary angiography may depict stenosis and saccular aneurysms involving one or more coronary arteries. There are no pathognomonic laboratory tests for Behcet’s disease, and it is best diagnosed in the context of recurrent oral ulcers, genital lesions, ocular lesions, and characteristic systemic involvements.^14^ Skin pathergy test that detects hypersensitivity response to needle-induced trauma is specific but less sensitive and can help in the diagnosis of Behcet’s disease. Polyarteritis nodosa is a systemic necrotizing vasculitis of medium arteries, and it is characterized by the absence of small vessel involvement.^15^ Multiple small fusiform or saccular aneurysms in renal, hepatic, and mesenteric arteries are well-recognized vascular complications. Coronary arteries are rarely involved. Asymmetric polyneuropathy causing motor and sensory loss and skin lesions like purpura, livedo reticularis, and ulcers are notable symptoms. It usually manifests in the 5th or 6th decade of life and affects both genders equally. Polyarteritis nodosa is distinguished from ANCA-associated vasculitis by a negative ANCA test. There is no diagnostic laboratory test for PAN and a definite diagnosis requires biopsy of the clinically affected organ. ANCA-associated vasculitis like granulomatosis with polyangiitis and microscopic polyangiitis are predominantly small vessel vasculitis, associated with ANCA and have similar renal involvement (focal necrotizing, often crescentic glomerulonephritis). They commonly present with non-specific symptoms like fever, malaise, anorexia, weight loss, myalgias, and arthralgias. Other common symptoms include urinary (haematuria, proteinuria, active urinary sediments), neurologic abnormalities (wrist or foot drop), renal impairment, and purpuric skin lesions. Cardiac involvement involving coronary aneurysm is rare. Diagnosis is confirmed by biopsy of the actively involved organs. Takayasu arteritis and giant cell arteritis are large vessel vasculitis that affects young to middle-aged and older women, respectively. Although aorta and branch vessels are most involved, medium vessels like coronary and pulmonary arteries may also be affected. Patients usually present with prolonged fluctuating constitutional symptoms (fever, weight loss, fatigue) in the early phase and later develop symptoms associated with vascular damage like pulselessness, claudication, light-headedness, syncope, hypertension, and gastrointestinal symptoms. Diagnosis can be made by clinical symptoms, presence of absent peripheral pulses, arterial bruits, elevated inflammatory markers like erythrocyte sedimentation rate, and C-reactive protein, and presence of arteritis in magnetic resonance angiography or CT angiography or PET scan. Notable non-inflammatory causes of CAAs are congenital CAAs and connective tissue disorders such as Ehlers–Danlos syndrome and Marfan syndrome. The reported incidence of congenital CAAs varies from 1% to 30%.^16^ They may be associated with other coronary anomalies like coronary artery fistula. Most patients remain asymptomatic; however, angina and breathlessness may be present secondary to luminal thrombosis and narrowing. Genetically inherited connective tissue disorders such as Marfan syndrome, and Ehlers–Danlos syndrome are characterized by abnormalities of the extracellular matrix disrupting the integrity of the vessel walls. Aorta is the most involved artery and medium-sized arteries like coronary arteries may also be involved. Clinical findings such as progressively dilated ascending aorta, aortic dissection, aortic regurgitation, mitral valve prolapse, excess linear growth of the long bones, tall stature, laxity of joints, arachnodactyly, pectus deformities, abnormal upper segment/lower segment, and arm span/height, ectopia lentis indicate Marfan syndrome. Ehlers–Danlos syndrome can be suspected from the presence of joint hypermobility, multiple joint dislocations, translucent skin, poor wound healing, easy bruising, unusual scars, aortic aneurysm, aortic dissection, mitral valve prolapse, myopia, and retinal detachment.^17^ Definite diagnosis of both the connective tissue disorders requires genetic testing of various components of extracellular matrix like collagen, fibrillin-1, and transforming growth factor (TGF) beta receptors 1 and 2.

The standard medical treatment of IgG4-related disease is immunosuppression with steroids. However, till date, there is no guidance from ESC recommendations on management of IgG4-related disease. In the early inflammatory phase, it is generally steroid responsive but as the disease progresses to the fibrotic phase, the response decreases. Also, there is a concern of aneurysmal rupture after steroid initiation as the pseudotumor surrounding the vessel wall and the thickness of the vessel wall decreases.^7^ B-cell depleting agent rituximab has shown some benefit in recurrent or relapse cases.^7,18^ The use of other immunosuppressants like methotrexate, azathioprine, and cyclophosphamide requires further evaluation. The endovascular treatment of CAAs includes covered stents and endovascular coiling. The surgical technique includes aneurysmectomy, thrombectomy, and aneurysmal ligation with or without bypass grafts. Generally, for left main involvement, multiple or giant coronary aneurysms, surgery is preferred.^19,20^

## Conclusion

IgG4-related disease presenting as an isolated CAA without other vessel involvement is rare. Although atherosclerosis remains by far the most common cause of CAA, the possibility of other aetiologies should be considered in appropriate settings. Multimodality imaging and a multidisciplinary team are required for the diagnosis and management of IgG4-related diseases.

## Supplementary Material

ytae028_Supplementary_Data

## Data Availability

The data underlying this article will be shared on reasonable request to the corresponding author.

## Lead author biography

The lead author of this case report is Rakavi. R. She is from Tamil Nadu, India. Currently, she is senior resident in the Department of Cardiology, All India Institute of Medical Sciences, New Delhi. She obtained her MBBS degree from All India Institute of Medical Sciences, Bhubaneswar, India and her MD internal medicine from Post Graduate Institute of Medical Education and Research, Chandigarh, India. The career objective of the author is work in the field of heart failure and research in cardiac pharmacology.
